# Intrinsic Functional Connectivity is Organized as Three Interdependent Gradients

**DOI:** 10.1038/s41598-019-51793-7

**Published:** 2019-11-04

**Authors:** Jiahe Zhang, Olamide Abiose, Yuta Katsumi, Alexandra Touroutoglou, Bradford C. Dickerson, Lisa Feldman Barrett

**Affiliations:** 1 Department of Psychology, Northeastern University, Boston, MA 02115 USA; 2 Center for Law, Brain and Behavior, Massachusetts General Hospital, Boston, MA 02114 USA; 3 Athinoula A. Martinos Center for Biomedical Imaging, Massachusetts General Hospital and Harvard Medical School, 149 13th St., Charlestown, MA 02129 USA; 4 Department of Neurology, Massachusetts General Hospital and Harvard Medical School, 149 13th St., Charlestown, MA 02129 USA; 5 Department of Psychiatry, Massachusetts General Hospital and Harvard Medical School, 149 13th St., Charlestown, MA 02129 USA

**Keywords:** Neuroscience, Neural circuits

## Abstract

The intrinsic functional architecture of the brain supports moment-to-moment maintenance of an internal model of the world. We hypothesized and found three interdependent architectural gradients underlying the organization of intrinsic functional connectivity within the human cerebral cortex. We used resting state fMRI data from two samples of healthy young adults (*N*’s = 280 and 270) to generate functional connectivity maps of 109 seeds culled from published research, estimated their pairwise similarities, and multidimensionally scaled the resulting similarity matrix. We discovered an optimal three-dimensional solution, accounting for 98% of the variance within the similarity matrix. The three dimensions corresponded to three gradients, which spatially correlate with two functional features (external vs. internal sources of information; content representation vs. attentional modulation) and one structural feature (anatomically central vs. peripheral) of the brain. Remapping the three dimensions into coordinate space revealed that the connectivity maps were organized in a circumplex structure, indicating that the organization of intrinsic connectivity is jointly guided by graded changes along all three dimensions. Our findings emphasize coordination between multiple, continuous functional and anatomical gradients, and are consistent with the emerging predictive coding perspective.

## Introduction

The brain has been described as an internal model of the world, dynamically constructing simulations from generative combinations of prior experience^1–3^. Intrinsic connectivity networks – ensembles of widely distributed brain regions with statistically dependent fluctuations in activity over time^4–6^ – are hypothesized to play a pivotal role in implementing and adjusting this model^7–15^. A *parcellation* approach to intrinsic connectivity networks assumes they are spatially discrete (i.e., modules) within the brain, such that each region belongs to one and only one network (e.g.^16–26^). Yet brain regions often show fluid coupling with different networks^27–29^, sometimes conceptualized as affiliating with multiple intersecting networks (e.g.^30–32^), and some networks play a more central role in the brain’s internal model than do others (e.g.^10^). A *connectomics* approach treats neural ensembles as overlapping sub-networks (e.g.^33,34^) that implement and update the internal model by communicating via densely connected “rich club” hub regions^35,36^. Connectomics does not, by itself, identify organizational features reflecting the brain’s ongoing activity (but see^37^ for a recent example of a connectomics approach that makes functional inferences). A *cytoarchitecture* approach provides additional computational insights by positing that the relative differences in cortical lamination in two connected cortical regions strongly predicts the type of information flow between those regions (e.g.^38,39^). When integrated with the principles of *predictive processing*^1,2,40^, this approach suggests specific hypotheses for the role that intrinsic connectivity networks play in maintaining and updating the brain’s internal model, the key hypothesis being that the internal model (called predictions) and learning signals (called prediction errors) propagate across neurons arranged in a loose hierarchy (see also^41^), with internal representations originating in limbic cortices, including agranular cortices that lack a well-defined layer IV and have sparser layers II and III, as well as dysgranular cortices with a rudimentary layer IV, such as the cingulate cortex, anterior insula and medial orbitofrontal cortex^38^. Moreover, via their projections to subcortical regions that regulate the autonomic nervous system and other systems of the internal milieu of the body (e.g., the hypothalamus, amygdala, ventral striatum, periaqueductal gray, parabrachial nucleus, nucleus of the solitary tract), limbic cortices are anatomically well-positioned to integrate information in the service of generating more efficient and accurate internal representations^11,32^. A crucial biological insight from the cytoarchitecture approach is that information flow in the cortex proceeds across continuous hierarchies. Notably, gradient-based approaches have also been successfully used to investigate cortical cell content^42^, thickness^43^, myelin content^44^, and genetic expression^45–47^. Therefore, across multiple levels of analysis, gradient-based approaches have facilitated investigations into the organizational patterns of brain structure and function.

In the current study, we integrated insights from the parcellation, connectomics, and cytoarchitectural approaches to develop a unified framework for describing the organizational features of intrinsic connectivity across the cerebral cortex. Specifically, we tested the hypothesis that intrinsic connectivity within the cortex is organized as *interdependent gradients* by which connectivity patterns show continuous similarity rather than discrete differences. Our approach emphasizes region-to-region affiliations in intrinsic connectivity (i.e., forming similarity gradients), which are largely ignored by the parcellation approach that focuses primarily on defining unique region-to-network affiliations. Our gradient-based analysis discovers how the connectivity of cortical regions shifts with changes in their location along several cortical hierarchies. Most importantly, our demonstration that intrinsic connectivity is organized on interdependent gradients is particularly novel, given that previous studies examining gradient-based cortical organization tended to treat connectivity gradients as statistically independent properties of the brain (e.g.^48–55^).

To sample intrinsic connectivity across the cortical sheet, we selected 109 seed regions across five intrinsic connectivity network motifs (recurring topographical patterns) most commonly identified in literature (Fig. S1 and Table S1) and estimated their intrinsic connectivity maps. This was done for two samples of participants (discovery sample *N* = 280, replication sample *N* = 270). For each seed, a group-level intrinsic connectivity map was computed and was used to generate a 109 × 109 similarity matrix with *η*^2 ^^56^ as an index summarizing pairwise similarity between intrinsic connectivity maps (Fig. S2). We discovered the organizing properties within this similarity matrix using multidimensional scaling (MDS)^57^. Briefly, MDS produces a quantitative dimensional description of the underlying structure of the data and maps it to a Euclidean coordinate space while preserving the pairwise similarities between data points; closer proximity in the remapped space indicates higher similarity. MDS confers several advantages over other techniques, such as principal component analysis (PCA), in understanding intrinsic connectivity organization: (1) MDS does not assume but can discover whether intrinsic connectivity neatly decomposes into non-overlapping components and (2) MDS tends to yield fewer, more interpretable dimensions than PCA^58^.

MDS allows a geometric depiction of the similarity matrix, which was important because we predicted that similarities between intrinsic connectivity maps would be represented as a circular array referred to as a *circumplex*^59^ (Fig. S3A). A circumplex pattern would indicate that network similarities exist in a continuous rather than a discrete fashion, the latter of which involves connectivity maps clustering in certain parts of the N-dimensional space but not in others (Fig. S3B; e.g., a strict discrete parcellation scheme with high within-cluster similarity and between-cluster difference). A circumplex would also suggest that similarity among maps can be described using more than one feature (i.e., the similarity is heterogeneous – that is, two intrinsic connectivity maps compared using a single gradient would be incomplete because their similarities are simultaneously described by multiple, interdependent gradients reflecting multiple descriptive features^60^ rather than by uncorrelated, additive gradients (Fig. S3C; e.g., each gradient uniquely explains a functional domain, and knowing the affiliation of a brain region with one domain would reveal nothing about how the same region’s affiliation with another domain). These two non-circumplex alternative cases are referred to as *simple structures*^61^.

## Results

### Goodness-of-Fit

**Figure 1:**
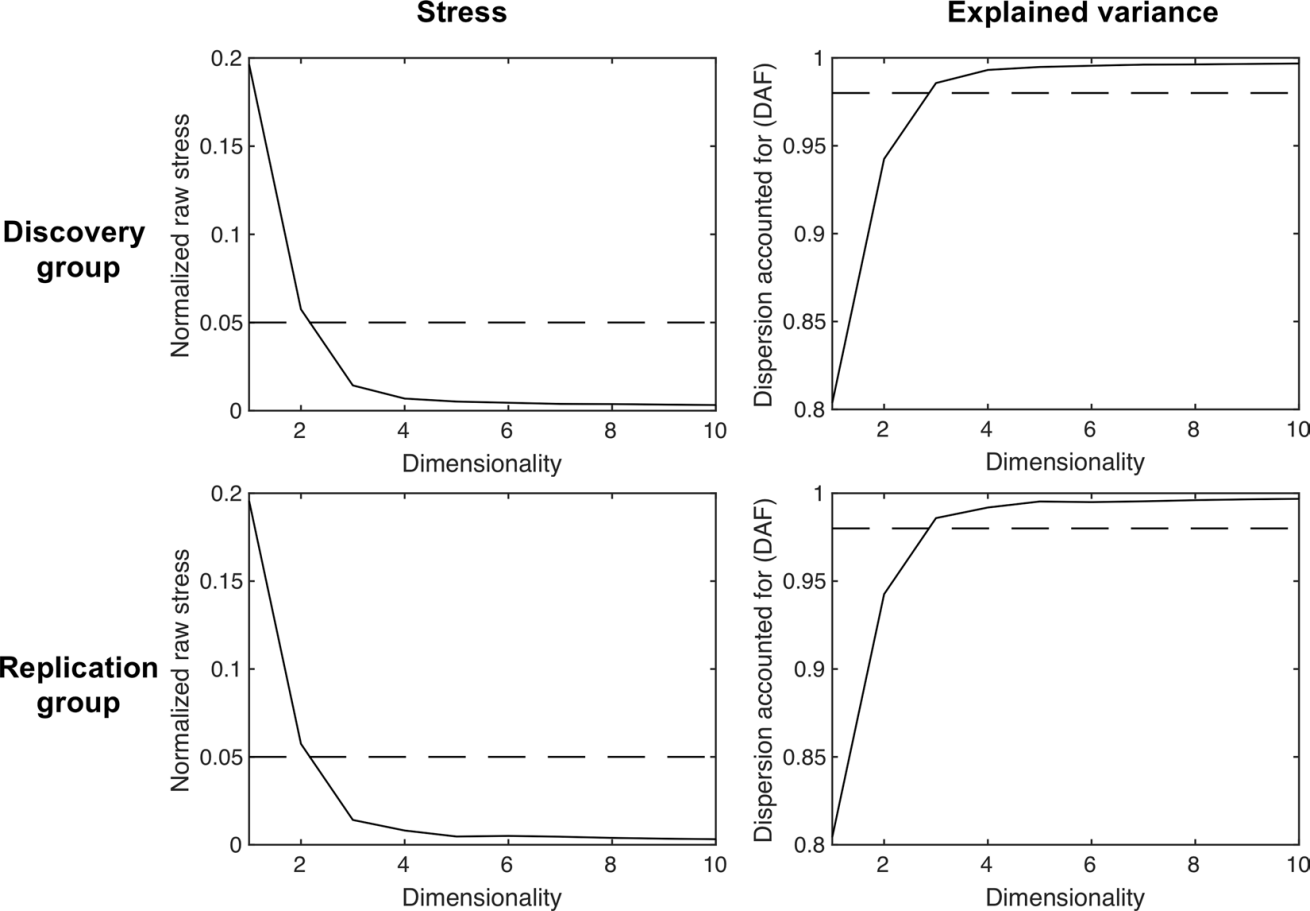
Both goodness-of-fit estimates were highly replicable across the discovery and replication groups and suggested that a three-dimensional solution was optimal. Stress was plotted as a function of the number of estimated dimensions. Lower stress indicates better fit. The scree plot of normalized stress had an “elbow” when dimensionality was at 3, since further addition of dimensions did not substantially reduce normalized stress. Dashed line indicates normalized stress of 0.05. DAF was plotted as a function of the number of estimated dimensions. Higher DAF indicates better fit. Dashed line indicates DAF of 0.98.

Stress and explained variance indicated that a three-dimensional solution was optimal to describe the similarities among the intrinsic connectivity maps in both discovery and replication samples (Fig. 1). A three-dimensional solution brought normalized stress below 0.05^57^ and captured over 98% of the variance^58^. All reported findings in the following sections were obtained using the discovery sample; similar results identified with the replication sample are reported in the SI (Figs S4–6). The three-dimensional solution remained optimal when we removed global signal regression from preprocessing, and also when we uniformly sampled 264 seeds across the cortex per^17^ (Fig. S7), indicating that the three-dimensional solution was robust to variations in preprocessing and seed definition.

### Circumplexity

**Figure 2:**
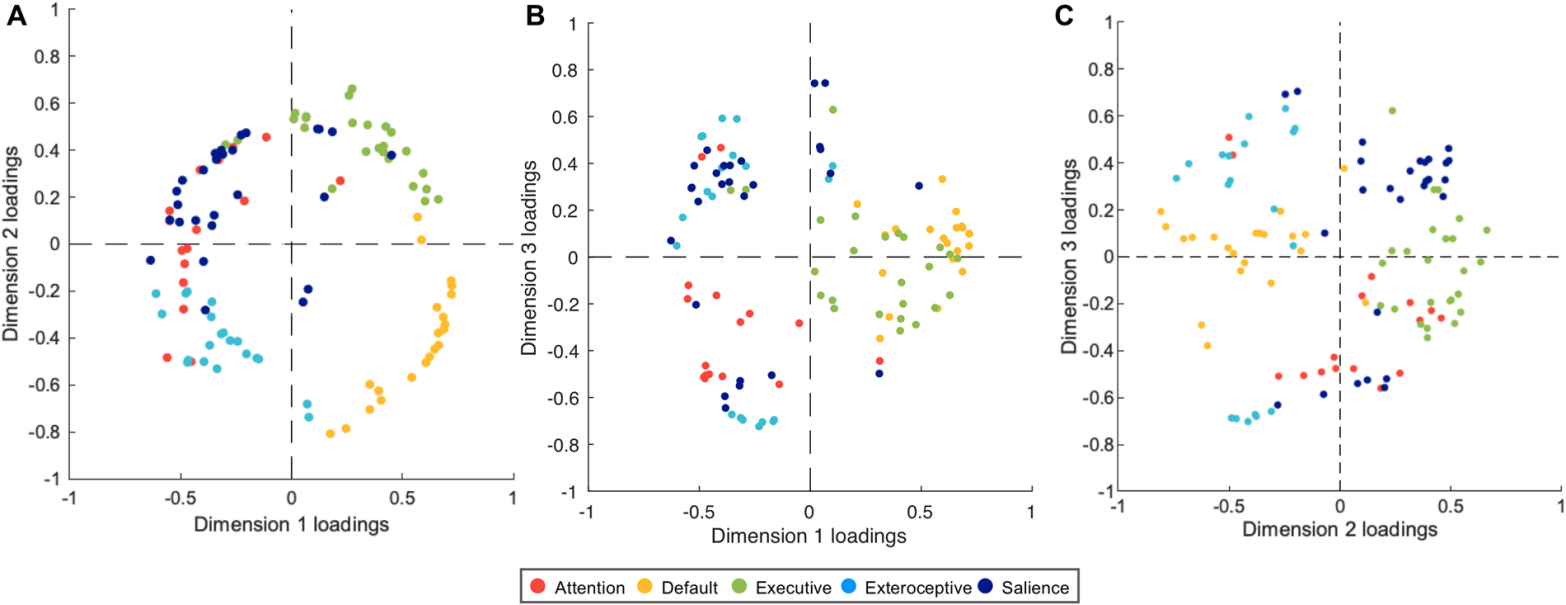
MDS results revealed that intrinsic connectivity patterns followed a circumplex structure of similarity. We calculated intrinsic connectivity maps based on 109 seeds across five most frequently identified network motifs in the published literature: attention (red), default mode (yellow), executive (green), exteroceptive (light blue), and salience (dark blue). Each point in the scatterplot represents a connectivity map. We plotted **(A**) Dimension 1 vs. Dimension 2, (**B**) Dimension 1 vs. Dimension 3, and (**C**) Dimension 2 vs Dimension 3 to facilitate interpretation.

We plotted the dimension loadings (ranging between −1 and 1) associated with all 109 intrinsic connectivity maps in Fig. 2. As predicted, the similarity between connectivity maps displayed circumplex behavior, i.e., the maps arrayed in a circular formation rather than clustering in particular parts of the Euclidean space. In other words, the literature-based motifs were not distinct modules and instead showed graded similarity in a three-dimensional Euclidean space. According to definitions of a circumplex, (1) there should be no preferred rotation of the dimensions that anchor the structure^62,63^ and (2) all variables should have a constant radius from the center of the circle^59,64,65^. Statistics based on these two criteria suggest that the solution derived from our data can be described as a circumplex. First, there was no preferred rotational solution for the results, consistent with what would be observed in a true circumplex structure per^66,67^ (Rotation Test: *RT* = 0.01, *p* < 0.01; Variance Test: *VT*2 = 0.12, *p* < 0.01). Second, the maps had a mean distance of 0.69 from the center, with a standard deviation of 0.09. The Fisher Test FT^66,67^; computed as coefficient of variation (the ratio of the standard deviation to the mean), was 12.79%, indicating that the maps varied within 6.5% on each side of the 0.69-radius circle. This variation was within the range reported for circumplex structures in previous literature^64^. As expected, we obtained similar circumplex structure when sampling 264 seeds (*RT* = 0.06, *p* < 0.01; *VT2* = 0.03, *p* < 0.01; *FT* = 11.45%), suggesting that the circumplex solution was robust to variations in seed definition (Fig. S8). Taken together, these findings, along with visual inspection of the solution, suggest that our solution indeed reveals circumplex features.

Within this circumplex organization, connectivity maps seeded in the same motif were closer together in Euclidean space (Fig. 2) and showed graded degrees of similarity with connectivity maps belonging to other motifs. For example, in Fig. 2A, maps that were seeded in the salience motif (dark blue; e.g., anterior insula, anterior cingulate cortex and supramarginal gyrus) overlapped with those seeded in the executive (green; e.g., middle frontal gyrus and inferior parietal lobule) and attention motifs (red; e.g., frontal eye field and superior parietal lobule). In fact, all neighboring motifs shared some overlap except the maps seeded in the default mode motif (yellow; e.g., medial prefrontal cortex, posterior cingulate cortex, dorsolateral prefrontal cortex, angular gyrus, temporal pole, and lateral temporal cortex). In Fig. 2B,C, the lack of motif boundaries was even more prominent; default mode (yellow) and executive (green) motifs completely overlapped in Fig. 2B. Interestingly, connectivity maps within attention (red), exteroceptive (light blue) and salience (dark blue) motifs also showed large variability along Dimension 3. For instance, some regions of the salience motif (e.g., cingulo-opercular regions) had high loadings on Dimension 3, whereas others regions of the same motif (e.g., frontoparietal regions) had low loadings on Dimension 3 (Fig. 2B,C).

**Figure 3:**
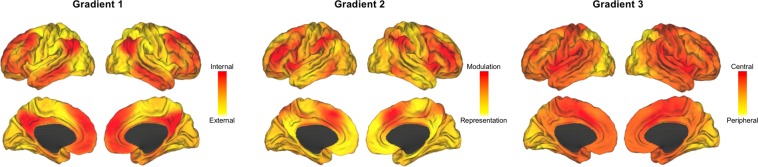
Gradient maps visualized on the brain surfaces. For each dimension, we created a gradient map by weighting every connectivity map by its dimension loading and summing across all weighted maps to create a composite (i.e., a weighted sum akin to factor scores). Gradient 1 (external vs. internal) captured a functional contrast between processing information from the external environment and the internal milieu. Gradient 2 (modulation vs. representation) captured a functional contrast between attentional modulation and content representation. Gradient 3 (anatomical centrality) captured a structural contrast between spatially central nodes and peripheral nodes. We visualized the gradient maps on inflated brain surfaces using Caret^154^.

As befits a circumplex, variation along one dimension was accompanied by variation along the others, indicating the interdependence of the features represented by those dimensions^60^. For example, in Fig. 2A, as loadings for connectivity maps seeded in the executive (green) and salience (dark blue) motifs decreased on Dimension 2, the former transitioned from zero to positive loadings on Dimension 1 while the latter transitioned from zero to negative loadings on Dimension 1. Therefore network motifs can be compared and contrasted based on how their loadings differently co-varied on all three dimensions. For example, when comparing between regions belonging to the default (yellow) vs. executive (green) motifs, we observe that both exhibit similar graded changes in Dimension 1 and Dimension 3 (Fig. 2B,C), however they exhibit remarkable differences in their involvement in Dimension 2 (Fig. 2A,C).

### Dimension interpretation

To determine the architectural gradients associated with the MDS dimensions, we created three “gradient maps” (these maps included subcortical components, which are not discussed here because they are not directly relevant to the predictive processing framework). For each dimension, we first multiplied every connectivity map by its corresponding dimension loading to create weighted maps and then summed across all weighted maps to create a final composite gradient map (i.e., a weighted sum akin to factor scores). In this way, each map’s contribution to a gradient map depended on how strongly it related to the MDS dimension. The summary gradient maps in Fig. 3 provide complementary interpretational value to the scatterplots in Fig. 2 because we can directly examine the relationship between regions and gradients, bypassing the literature-based motif categories (to avoid confusion, we refer to values on MDS dimensions as dimension ‘loadings’ and values on gradient maps as gradient ‘scores’). The brain gradients captured three features of cortical architecture (Fig. 3).

Gradient 1 corresponded to an *external vs. internal gradient*, replicating^51^. Regions with lower scores on Gradient 1 belonged with motifs that are relatively important for representing signals that are external to the brain, such as the exteroceptive sensory motif (e.g., visual, auditory or sensorimotor networks; e.g.^68^), as well as motifs that are important for modulating the representations of those signals - e.g., the dorsal attention network^69^ and the salience motif consisting of the cingulo-opercular^70^, multimodal^71^, salience^72^ or ventral attention^69^ networks. Regions with higher scores on Gradient 1 belonged with motifs that are relatively important for constructing and maintaining the representations that constitute the brain’s internal model, such as the default mode^73^ and mentalizing^74^ networks, and motifs that are important for modulating those representations - e.g., executive control^72^ or multiple-demand^75^ networks.

Gradient 2 corresponded to a *representation vs. modulation gradient*. Regions with lower scores on Gradient 2 belonged with motifs that are important for representing mental content, such as sensory information in the primary sensory cortices and multimodal summaries of brain states in the default mode network^68^. Regions with higher scores on Gradient 2 belonged with modulation-related motifs (any process that operates on sensory or motor representations, such as attention regulation, goal maintenance, strategy selection, performance monitoring), such as the executive control^76^ and salience^77^ motifs.

**Figure 4:**
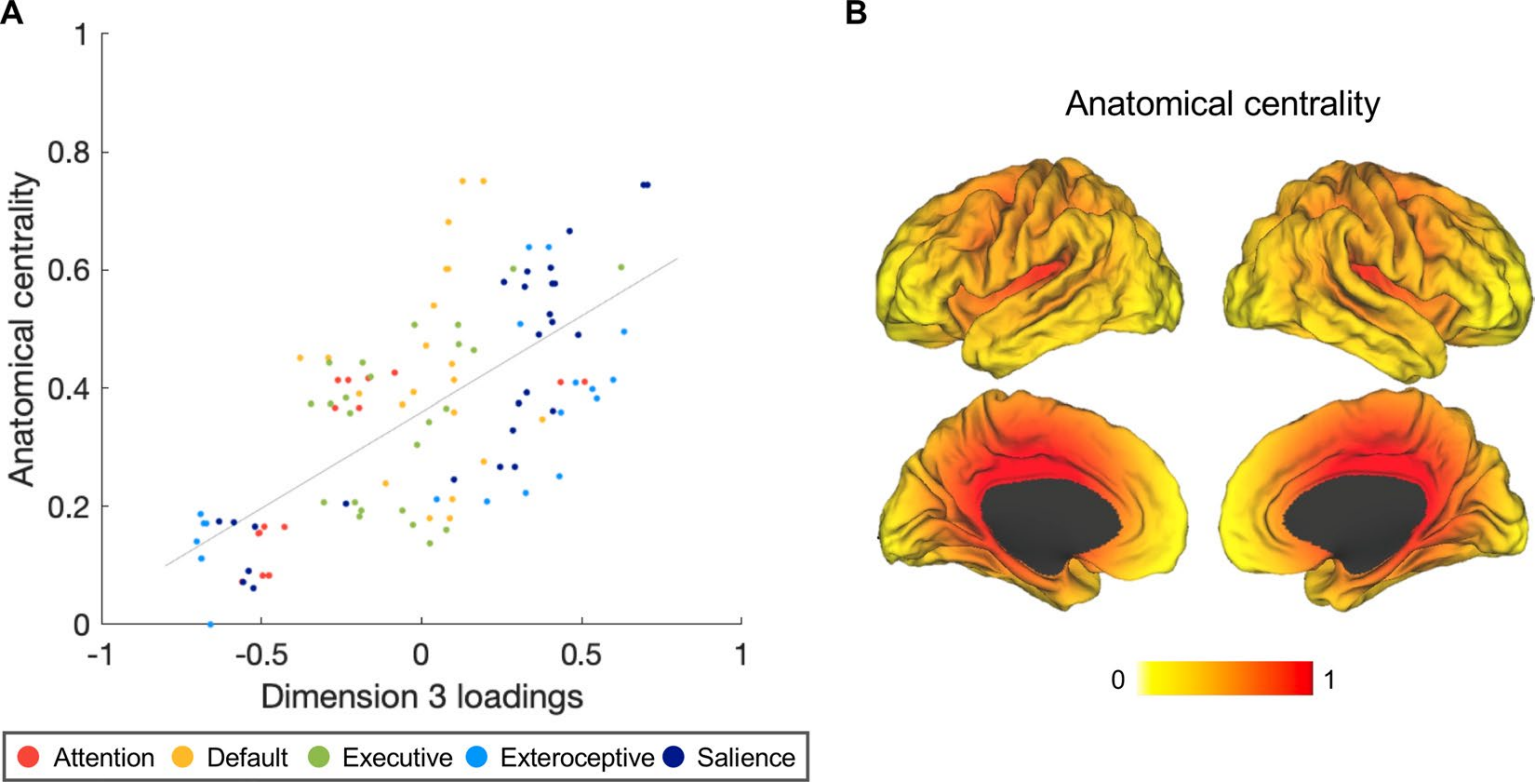
Gradient 3 corresponded to anatomical centrality. (**A**) Dimension 3 loadings correlated positively with anatomical centrality (*N* = 109; *r*(107) = 0.676, *p* < 0.001). (**B**) Anatomical centrality for each seed was computed as (maximal distance – node distance)/maximal distance, where maximal distance is the distance for the node with maximal distance from the anterior commissure, with 0 indicating minimum anatomical centrality (at maximum distance from the anterior commissure) and 1 indicating maximum centrality (at the anterior commissure). The anterior commissure was used as a proxy for the center of the brain since it is approximately equidistant from the most distal points of the cerebrum. We visualized anatomical centrality values on inflated brain surfaces using Caret^154^.

Gradient 3 corresponded to *anatomical centrality* (geometric term describing lack of Euclidean distance from center of the brain^78^). Regions with lower scores on Gradient 3 were more peripheral in the cortex, including the primary visual cortex, lateral frontal and lateral parietal regions. Regions with higher scores on Gradient 3 occupied anatomically more central positions in the cortex. We empirically tested this gradient by correlating Dimension 3 loadings of the connectivity maps with anatomical centrality values of corresponding seeds (computed as normalized proximity to anterior commissure; Fig. S9, see detailed description in SI). We found a strong, positive association (Fig. 4A; *r* = 0.676, *p* < 0.001), suggesting that seeds located closer to the anatomical center of the brain tended to anchor intrinsic connectivity maps with higher loadings on Dimension 3. Anatomical centrality (Fig. 4B) can be considered a proxy for laminar differentiation^78^ since cortices become progressively laminated as one moves from the central limbic cortices (forming a ring around the corpus callosum and defining the limits of each hemisphere, including anterior to midcingulate cortex, anterior insula, and temporal pole) towards the periphery of the cortex (see^38^^,79^;).

**Figure 5:**
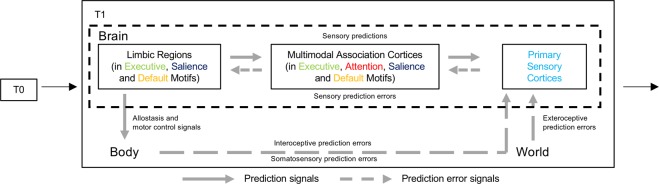
Predictive processing framework. Starting with initial conditions in the body and in the world (T0), the brain is thought to continually predict forward in time (T1), preparing changes in the body’s internal systems to support upcoming motor actions. Efferent copies of these motor and visceromotor preparations function as their predicted sensory consequences, cascading to sensory systems to modulate the firing of sensory neurons in advance of incoming sensory inputs. Sensory inputs from the body and the world are continuously compared to prediction signals. If different, prediction errors are sent to update the brain’s internal model for future occasions. This framework is based on a structural model of cortico-cortical connections whereby predictions flow from less to more laminated (i.e., layered) cortices (‘feedback connections’), whereas prediction errors flow in the opposite direction (‘feedforward connections’)^155,156^. This structural model is consistent with a gradient- but not module-based organization scheme for intrinsic connectivity. This is because the whole brain is thought to participate in predictive processing, not by separating into mental modules, but by operating on a continuous two-way hierarchy. In the feedback direction, abstract predictions are unpacked into particular sensory simulations; at the same time in the feedforward direction, sensory information is compressed to be integrated with the brain’s internal model (see review in^7^). Figure adapted from^104^.

## Discussion

Intrinsic functional connectivity within the cerebral cortex can be described by three *interdependent* architectural gradients, which spatially correlate with two functional features (*external vs. internal* and *representation vs. modulation*) and one structural feature (*anatomically central vs. peripheral*) of the brain. When projected into geometric space, the similarities between connectivity maps were represented by a circumplex structure. This finding suggests that the organization of intrinsic functional connectivity shows continuous similarity across multiple, interdependent gradients rather than discrete differences. Overall, our findings highlight the importance of simultaneously considering functional and anatomical hierarchies in the brain, integrating parcellation, connectomics, and architectural approaches to understanding cortical function.

Our results are consistent with available evidence that intrinsic connectivity can be described with continuous local^48,53,55,80^ and global^51,54,81,82^ gradients, suggesting that intrinsic connectivity motifs do not constitute discrete networks. Prior research using the parcellation approach identified spatially discrete intrinsic connectivity networks by: (1) using methods that force independence (e.g., cluster analysis^16–20^), (2) setting arbitrary thresholds that dissociate networks (e.g.^69,83,84^), or (3) emphasizing independence rather than correlation between networks when using methods such as ICA (e.g.^85–88^). Although these techniques may be useful for certain purposes (e.g., to create heuristic parcellation schemes), their emphasis on assigning a unique network membership to each cortical region does not allow a fully realized interpretation of the organizing principles underlying intrinsic functional connectivity, which are actually based on continuous similarity gradients. This is an important limitation of the parcellation approach, because recent evidence shows that network motifs are, in fact, connected, and overlapping in rich club hubs^32^, which has functional implications (e.g.^8,89–91^). Allowing coupling across networks, for instance, helps identify functional connections that are crucial for task-dependent global integration^15,92^.

More importantly, we observed that the similarities between functional connectivity patterns were not just graded, but their variations were also interdependent across the different gradients, as suggested by the circumplex ordering of similarities. The MDS solution satisfied two circumplex criteria: no preferred rotation and constant radius. In an ideal circumplex, elements are arrayed in a circular fashion, so mathematically, there is not one rotational solution that best describes the data structure^59^. Note that while it is important to establish a lack of preferred rotation for quantifying circumplexity, it is also important to determine one set of dimensions that best represents the features underlying functional connectivity organization for interpretational purposes^60,93^. The set of three dimensions reported herein are consistent with other findings on structural and functional organization of the brain (detailed below), indicating that the observed three dimensions are valid and useful in characterizing the organization of intrinsic functional connectivity. Our discovery aligned with the hypothesized interdependency scenario (Fig. S3A). In contrast, if a discrete simple structure (Fig. S3B) were found, it would mean that intrinsic connectivity was organized in a fully modular fashion, where each domain was its own dimension and was unrelated to the other dimensions, as reflected by concentrated loadings on one end of the dimension only. If a non-discrete simple structure (Fig. S3C) were found, it would mean that dimensions were independent from each other, e.g., Gradient 1 (internal vs. external) and Gradient 2 (modulation vs. representation) do not covary. In other words, knowing that default mode regions scored high on internal-processing would tell us nothing about how they scored on the representation vs. modulation gradient.

Notably, our results are also robust across methodological variations. To test the optimal dimensionality and stability of the circumplex structure observed in our data, we varied preprocessing (global signal regression) and analytical (seed definition) parameters to see if they would disrupt ordinal orderings in the similarity matrix, which could lead to changes in the MDS solution^58^. We performed global signal regression because it is considered an effective means to reduce artifacts in resting state data^94^, and deviation scoring in general (removing mean signal in raw data) enhances statistical power in circumplexity tests by removing any potential general factor^66,67^. This technique is also known to artificially inflate negative correlations^95–98^ because it shifts the entire distribution of correlations in the negative direction^94^. We anticipated the ordering of pairwise similarities to be retained with or without the shift. In addition, we selected 109 canonical network seeds from published papers because they should yield maximal between-network differences and uncover simple structures in the data (Fig. S3B,C) if they did exist. Since these literature-based seeds did not identify simple structures, we expected that a more comprehensive sampling of 264 seeds would likely fill out the space within the circumplex structure. As expected, the optimal dimensionality and general circular ordering of similarities were not affected by changes in the preprocessing and analytical parameters, and global signal regression improved the detection of circumplexity. These additional analyses demonstrate the robustness of the circumplex organization across methodological variations and provide strong support to our hypothesis of multiple interdependent gradients.

To interpret these gradients, we turned to a novel predictive processing framework that depends on cytoarchitecture to understand the intrinsic organization of the cerebral cortex. This predictive processing framework has been used to study topics as wide-ranging as sensory and motor systems^99–101^, individual neuron dynamics^102^, brain energetics^103^, and consciousness (e.g.^7,9,11,100^). This framework is anchored by the hypothesis (outlined in^7,104^) that an animal’s cerebral cortex, the cerebellum (e.g.^105^) and the hippocampus (e.g.^106^) create an internal model of the animal’s body in the world, constantly using past experiences to anticipate the needs of the body in relation to predicted sensory inputs and preparations for motor action, and attempting to meet those needs before they arise, through a process called allostasis^107,108^ (see Fig. 5 for a schematic diagram of this framework).

The three gradients describing cortical intrinsic connectivity can be interpreted as capturing different components of the predictive processing framework. Gradient 1 describes a gradient that runs, at one end, from the motifs that are important for processing the sensory input that continually confirms or refines the internal model (low gradient scores) to, at the other end, the motifs that are important for generating the prediction signals that constitute the brain’s internal model (high gradient scores). Regions low on this gradient belonged with primary sensory, attention, and salience motifs, which are more associated with externally oriented processes such as sensory perception, goal-directed selection for stimuli^109^, processing relevance of personally salient sensory information^72^, and the integration of multisensory information from the periphery^71^. Regions high on this gradient belonged with default mode and executive control motifs, which are more associated with internally oriented processes such as mind-wandering, introspection, and autobiographical planning^110,111^. This functional contrast is also sometimes called ‘external’ versus ‘internal’ modes of cognition^112,113^, or ‘bottom-up’ versus ‘top-down’ processing^114^. It is similar to the ‘principal sensorimotor-to-transmodal gradient’ obtained using a different dimension reduction technique on resting state fMRI data^50,51^, which was also anchored on one end by primary sensorimotor regions and on the other end by the default mode network. This gradient has been identified in tract-tracing studies of non-human primates (macaque^51^, and marmoset^115^) and is consistent with cortical myelin gradient^49^ as well as with genetic transcription gradient^45–47,116^, suggesting that the brain’s microstructural integrity and genetic profile are implicated in the brain’s functional wiring. Our interpretation is also consistent with evidence from the connectomics literature showing that default mode regions (high gradient scores) are capable of steering the brain into different states with little input of energy – i.e., using information available from the internal model (average controllability^37^), whereas salience regions (low gradient scores) drive the brain into states that require more input of energy – i.e., learning or encoding (modal controllability^37^).

Gradient 2 distinguishes voxels that belong to regions that predominantly represent prediction and prediction error signals from those voxels that belong to regions that predominantly implement attentional modulation, or precision^117,118^. Regions low on this gradient belonged with motifs that are more associated with content representation. More specifically, the default mode regions represent multimodal summaries of brain states^68,119^ or supramodal conceptual knowledge^120^, and are hypothesized to represent the brain’s internal model, whereas the primary sensory regions represent sensory input from the external environment^68^ and from the body^7,121^. Regions high on this gradient belonged with executive control and salience motifs, which are thought to be involved in top-down modulation of the default mode and executive networks (e.g.^122,123^), and are hypothesized to tune the precision of predictions and prediction errors, respectively^7,121^. It is not surprising that regions with high scores on this gradient replicate the task positive network^124^ or multiple demand network^75^, because fMRI experimental tasks are typically designed to require attention modulation (e.g., randomized trials and jittered inter-trial intervals elicit more deliberate, controlled and effortful processing^104^). The attention motif occupied the middle portion of the gradient, consistent with its role in linking sensory information to motor responses^125^. This gradient appears similar to the third principal gradient reported in^51,126^, although the authors provided no interpretation of this dimension.

Gradient 3, like the previous two gradients, was computed based on functional connectivity. However, this third gradient appeared to represent anatomical centrality, which is a structural feature related to the systematic variation in the degree of cortical laminar differentiation. Specifically, limbic cortices form the spatial core of each hemisphere. Multimodal association regions (granular cortices, eulaminate I), followed by primary sensory regions (granular or koniocortices, eulaminate II), spatially irradiate from the core limbic areas and exhibit increasingly developed laminar structure (reviewed in^38,41,79,127–129^). Consistent with this pattern, limbic cortices scored highly on Gradient 3, whereas multimodal association regions, followed by primary sensory areas scored progressively lower on Gradient 3. To our knowledge, no published empirical study has identified this third gradient in describing the organization of intrinsic functional connectivity. Within the predictive processing framework, the spatial position of the limbic core is important for several reasons. Developmentally, limbic cortices form first and generate widespread feedback projections to other regions in the brain^38,130,131^. They are hypothesized to easily modify neural activity in eulaminate areas and promote functional flexibility via its diverse feedback connections^38^. Therefore, limbic cortices have been hypothesized to create a highly connected, dynamic functional ensemble for information integration and accessibility in the brain^11^. Adding to this literature, our current finding shows that the spatially central position of limbic cortices also has implications for the organization of intrinsic functional connectivity. Integrating information about the cytoarchitecture of the brain (i.e., laminar differentiation) into functional connectivity organization can help us understand known fractionation schemes of the default mode and salience motifs in the literature. The default mode motif has been found to fractionate into a relatively central subsystem (including medial limbic nodes such as the subgenual anterior cingulate cortex, retrosplenial cortex, parahippocampal gyrus, and the hippocampal formation) and a relatively peripheral subsystem (including more lateral nodes such as the temporal parietal junction, lateral temporal cortex, and temporal pole)^110^. Similarly, the salience motif has been found to consist of a more central subsystem (including limbic nodes such as the amygdala, ventral anterior insula, and pregenual anterior cingulate cortex) and a more peripheral subsystem (including more lateral nodes such as medial frontal gyrus and supramarginal gyrus)^84^.

The novel evidence reported here encourages future research on several aspects related to the organization of intrinsic functional connectivity based on multiple interdependent gradients. First, following prior work revealing task-related modulation of intrinsic connectivity^132^, it would be important for future research to investigate whether the same gradients emerge during task states. Such work would clarify whether the three interdependent gradients found in the current study are stable features of functional cortical architecture regardless of situational demands. Second, our analyses involved correlation of blood-oxygen-level dependent (BOLD) activation time courses during a whole resting state scan, but recent research on dynamic functional connectivity shows that the amount of coherence between regions could vary in a short time period^133,134^ and in longer-term development^135^, prompting the question of whether the three-gradient architecture withstands dynamic reconfigurations in functional coupling observed over time. Third, we calculated the connectivity similarity matrix on a group level and did not probe individual differences. Recent research revealed finer details of network fractionation that were only observable at the individual level^136^, suggesting that the distribution of regions on the similarity gradients may slightly shift from person to person, as individual differences in functional coupling may arise given distinct past experiences. Examination of individual variations in the gradient-based organization of intrinsic connectivity, therefore, would be a promising avenue for future research in identifying its role in complex behaviors and psychological phenomena. To this end, high resolution fMRI acquisition and voxelwise analysis technique may facilitate a more nuanced understanding of individual-specific variations in similarity gradients. Fourth, our measure of anatomical centrality, based on distance to the anterior commissure, is one proxy for the laminar differentiation gradient; other measures (e.g., neuronal density or myelin) have been proposed as well, although they contain important limitations in capturing laminar differentiation (see detailed discussion in^127^). Future studies might consider the advantages of other estimates of laminar differentiation. Finally, a few previous studies^43,51^ have examined distance in the brain using geodesic distance along the curvatures of the cortical mantle instead of Euclidean distance. Future investigations should systematically compare the similarities and differences in their abilities to predict connectivity and other brain characteristics.

## Materials and Methods

### Participants

Participants in this study were 660 healthy young adults (55% female, 18–30 years), previously described in^16,32,137,138^. All were native English-speakers with normal or corrected-to-normal vision and reported no history of neurological or psychiatric conditions. Experimental protocol was approved by the institutional review boards of Harvard University and Partners Healthcare. All research was performed in accordance with relevant guidelines and written informed consent was obtained from all participants. We removed 79 participants (11%) due to head motion and outlying voxel intensities, and 31 participants (4.7%) due to a lack of signal in superior and lateral parts of the brain (outside of acquisition field). Our final dataset of 550 participants was randomly divided into a discovery sample of *N* = 280 (62% female, 19.3 ± 1.4 years) and a replication sample of *N* = 270 (53% female, 22.3 ± 2.1 years).

### MRI and fMRI

Participants completed structural and resting-state MRI scans, as well as other tasks unrelated to the current analysis. MRI data were acquired at Harvard and the Massachusetts General Hospital across a series of matched 3T Tim Trio scanners (Siemens, Erlangen, Germany) using a 12-channel phased-array head coil. Structural data included a high-resolution multi-echo T1-weighted magnetization-prepared gradient-echo image (multi-echo MPRAGE). Parameters for the structural scan were as follows: repetition time (TR) = 2,200 ms, inversion time (TI) = 1,100 ms, echo time (TE) = 1.54 ms for image 1 to 7.01 ms for image 4, flip angle (FA) = 7°, voxel size 1.2 × 1.2 × 1.2 mm and field of view (FOV) = 230 mm. The resting state scan lasted 6.2 min (124 time points) and participants were instructed to remain still, stay awake, and keep their eyes open. The echo planar imaging (EPI) parameters for functional connectivity analyses were as follows: TR = 3,000 ms, TE = 30 ms, FA = 85°, voxel size 3 × 3 × 3 mm, FOV = 216 mm and 47 axial slices collected with interleaved acquisition and no gap between slices. To preprocess the resting state data, we removed first 4 volumes, corrected slice timing, corrected head motion, normalized to the MNI152 template, resampled to 2 mm cubic voxels, removed frequencies higher than 0.08 Hz, smoothed with a 6 mm FWHM kernel and did nuisance regression (six motion parameters, average global signal, average ventricular and white matter signals)^139–141^. We also preprocessed the same dataset without global signal regression (GSR) and obtained similar results (‘109 seeds, GSR−’; Figs S7 and S8).

### Selection of network seed regions

From the network-parcellation literature, we selected 109 seed regions across five intrinsic connectivity network motifs most commonly identified in the literature. The rationale for choosing these canonical network anchors was to derive the most distinctive connectivity patterns possible and maximize the possibility of finding simple structures in functional organization if they existed. The attention motif consisted of visual attention regions, also collectively referred to as dorsal attention network^16,69,83^. For the default mode motif, we sampled seeds from amygdala affiliation^142^, default mode^16,110^, language^143,144^, and mentalizing^74^ networks. For the executive control motif, we sampled seeds from executive^16,72,83^ and multiple-demand^75^ networks. For the exteroceptive motif, we sampled seeds from amygdala perception^142^, auditory^145–147^, sensorimotor^16,146–148^, and visual^16,145,146^ networks. For the salience motif, we sampled seeds from amygdala aversion^142^, cingulo-opercular^70^, multimodal^71^, salience^72,84^ and ventral attention^16,149^ networks. See seed locations in Fig. S1 and MNI coordinates in Table S1. Homogeneity of each seed was calculated as the average temporal correlation between all unique pairs of within-seed voxels following previous reports^18,81,150,151^. The mean (M) and standard deviation (SD) of seed homogeneity for each network motif was identified as follows: Attention (M = 0.800, SD = 0.019), default (M = 0.822, SD = 0.016), executive (M = 0.810, SD = 0.016), exteroceptive (M = 0.795, SD = 0.023), and salience (M = 0.797, SD = 0.016). Overall, these values are comparable to or even higher than those reported in previous studies of functional connectivity parcellations, suggesting that seed homogeneity in the present study was sufficiently high on average. Seed homogeneity varied between the network motifs (*F*(4,1116) = 169.41, *p* < 0.001, *η*_*p*_^*2*^ = 0.378), as previously shown (e.g.^81^). However, the observed seed heterogeneity was not relevant for testing our hypotheses because we focused on gradients examining all network motifs along a continuum rather than analyzing discrete, modular networks. Therefore, no further analyses were performed on this metric. We also sampled an alternative set of 264 seeds across the cortex^17^ and obtained similar results (‘264 seeds, GSR+’; Figs S7 and S8).

### Functional connectivity and similarity matrix calculation

We calculated group-level whole-brain intrinsic connectivity maps following established seed-based procedure^83,84^. For each seed, we created a 4 mm spherical region of interest (ROIs) and extracted the average time course of BOLD activity within the ROI. We computed Pearson’s product moment correlations, *r*, between the seed time course and all voxels across the brain, converted those *r* values to *z* values using Fisher’s *r*-to-*z* transformation, and averaged the resulting *z* map across all subjects within each sample to obtain two group intrinsic connectivity maps per seed (one for each sample). To determine which connectivity values were meaningful in a group map, we relied on replication, as guided by classical measurement theory^152^, instead of imposing an arbitrary *z* threshold. This prevents type I and type II errors, which are enhanced with the use of stringent statistical thresholds^153^. It was uniquely important for us to consider all meaningful connectivity, including weaker but replicable connectivity, because its inclusion reveals more accurate degrees of similarity between intrinsic connectivity maps, while its exclusion means stronger connectivity is given more weight in comparisons and therefore enhances differences between network motifs. We binarized both group intrinsic connectivity maps at *z* = 0 since the interpretation of negative correlations can be ambiguous^95,141^ and took the conjunction between the two samples. We then masked the original non-binarized group intrinsic connectivity maps using this conjunction map to retain strengths of all positive correlations that are reliable across both samples. This procedure was repeated for all 109 seeds. Finally, for each sample, we calculated a 109 × 109 similarity matrix (*η*^2 ^^56^) between all masked intrinsic connectivity maps. Functional connectivity and similarity matrix calculation is illustrated in Fig. S2. Note that we used the ‘replication sample’ both for determining which connectivity was meaningful and for showing replicable MDS results. To demonstrate that the high replicability observed in our results was not solely driven by the method of thresholding, we also thresholded functional connectivity at *z* = 0.2^84,142^ instead of using replication. As expected, these analyses yielded similar three-dimensional (Fig. S10) circumplex solutions (Fig. S11). Additionally, given that some anticorrelations may be meaningful^98^, we tested the effect of including negative connectivity below *z* = −0.2 as well. This analysis yielded replicable three-dimensional (Fig. S12) circumplex solutions (Fig. S13).

### MDS analysis

To model similarities in maps, we used the PROXCAL algorithm in SPSS 23 (www.ibm.com/DataStatistics/SPSS). For each sample, we used the 109 × 109 similarity matrix as input and tested model fit for dimensionalities between 1 and 10. We determined the optimal dimensionality using two goodness-of-fit estimates: stress and explained variance. Stress is the square root of a normalized ‘residual sum of squared’. Higher stress indicates worse fit. Optimal dimensionality often manifests as the elbow of the stress plot and brings stress below 0.05^57^. The measure we used for explained variance is dispersion accounted for (DAF), which is equivalent to squared Tucker’s coefficient of congruence. Both goodness-of-fit estimates across the two samples indicated 3 was the optimal dimensionality (Fig. 1). Therefore, as output of the MDS analysis, we obtained 3 sets of 109 dimension loadings for each sample.

### Circumplexity evaluation

According to definitions of a circumplex, (1) there should be no preferred rotation of the dimensions that anchor the structure^62,63^ and (2) all variables should have a constant radius from the center of the circle^59,64,65^. We quantified the degree of circumplexity in the three-dimensional MDS solution using these two criteria, employing the Rotation or Variance Test, and the Fisher Test respectively^66,67^. The Rotation Test assesses the degree to which different rotations of the dimensions affects the solution. In addition, we also used the Variance Test (VT2^66^ to assess the impact of rotation by measuring the coefficient of variation in the amount of variables that fall in between any orthogonal pair of axes. We compared the test statistics of the Rotation and Variance Tests to the critical values reported in^66,67^ to determine the likelihood that our solution achieved the formal criteria for circumplexity. The Fisher Test assesses the degree to which the elements array in constant radius by measuring the coefficient of variation (i.e., the ratio of the standard deviation to the mean) in vector length^64^. When testing the circumplexity in the published literature in personality and affect data, the data are first deviation scored (i.e., each raw score is subtracted from the subject’s mean score^66,67^). Since global signal regression at the individual subject level is comparable to deviation scoring, we only tested circumplexity of MDS results using data on which global signal regression was performed during preprocessing.

### Anatomical centrality estimation

To estimate anatomical centrality, we used the anterior commissure as a proxy for the center point of the brain, since it is approximately equidistant from the most distal points of the cerebrum on the x, y, and z axes, and approximately occupies the middle point along the y axis of the medial limbic ring consisting of the cingulate cortex and medial orbitofrontal cortex (Fig. S9). We calculated the Euclidean distance between each seed (MNI x, y, z) and the anterior commissure (MNI 0, 0, 0) ($\sqrt{({x}^{2}+{y}^{2}+{z}^{2})}$) and computed a normalized measure of anatomical centrality for each seed defined as (maximal distance – node distance)/maximal distance, where maximal distance is the maximal distance from the anterior commissure to a seed, so that the anterior commissure would have an anatomical centrality of 1 and the most distant seed would have an anatomical centrality of 0. Anatomical centrality has been shown to be related to the degree of laminar differentiation and predictive of topological organization in the cortex^78^.

## Supplementary information

Supplementary Information

## Data Availability

The data that support the findings of this study are available as part of the Brain Genomics Superstruct Project (https://www.neuroinfo.org/gsp138). Gradients maps are available at: https://neurovault.org/collections/5449/.
