# Supplementary material for: Intrinsic Functional Connectivity is Organized as Three Interdependent Gradients
Source: Sci Rep. 2019 Nov 4;9:15976. doi: 10.1038/s41598-019-51793-7 (PMC6828953; doi:10.1038/s41598-019-51793-7)
Supplement: Supplementary file 1 — Supplementary Information [file 41598_2019_51793_MOESM1_ESM.docx]

**Intrinsic Functional Connectivity is Organized as Three Interdependent Gradients**

Jiahe Zhang^a1^, Olamide Abiose^b1^, Yuta Katsumi^a^, Alexandra Touroutoglou^c,d,e^, Bradford C. Dickerson^c,d,e^ & Lisa Feldman Barrett^a,c,d*^

^a^Department of Psychology, Northeastern University, Boston, MA 02115

^b^Center for Law, Brain and Behavior, Massachusetts General Hospital, Boston, MA 02114

^c^Athinoula A. Martinos Center for Biomedical Imaging, Massachusetts General Hospital and Harvard Medical School, 149 13^th^ St., Charlestown, MA 02129

^d^Department of Neurology, Massachusetts General Hospital and Harvard Medical School, 149 13th St., Charlestown, MA 02129

^e^Frontotemporal Disorders Unit, Department of Neurology, Massachusetts General Hospital and Harvard Medical School, 149 13th St., Charlestown, MA 02129

^1^J.Z. and O.A. contributed equally to this work.

**Supplementary Information**

Figure S1. We sampled 109 seeds based on previous literature. These seeds span 5 major motifs: attention (red), default (yellow), executive (green), exteroceptive (light blue), and salience (dark blue). Note seeds belonging to different motifs sometimes occupy the same anatomical region, e.g. attention (red) and salience (dark blue) in superior parietal lobule. We visualized the seeds on inflated brain surfaces using the BrainNet Viewer^1^.

Figure S2. Flowchart for functional connectivity and similarity matrix calculation. We relied on replication instead of arbitrary threshold to find meaningful connectivity.

Figure S3. Illustration of possible MDS results. A) Circumplex. B) Discrete simple structure. C) Non-discrete simple structure.

Figure S4. MDS results of the *replication sample* also revealed a circumplex structure of similarity. Each point in the scatterplot represents a connectivity map. We plotted A) Dimension 1 vs. Dimension 2, B) Dimension 1 vs. Dimension 3, and C) Dimension 2 vs. Dimension 3 to facilitate interpretation. *N* = 109.

Figure S5. Gradient maps of the *replication sample* visualized on the brain surfaces. For each dimension, we created a gradient map by weighting every connectivity map by its dimension loading and summing across all weighted maps to create a composite (i.e., a weighted sum akin to factor scores). We visualized the gradient maps on inflated brain surfaces using Caret^2^.


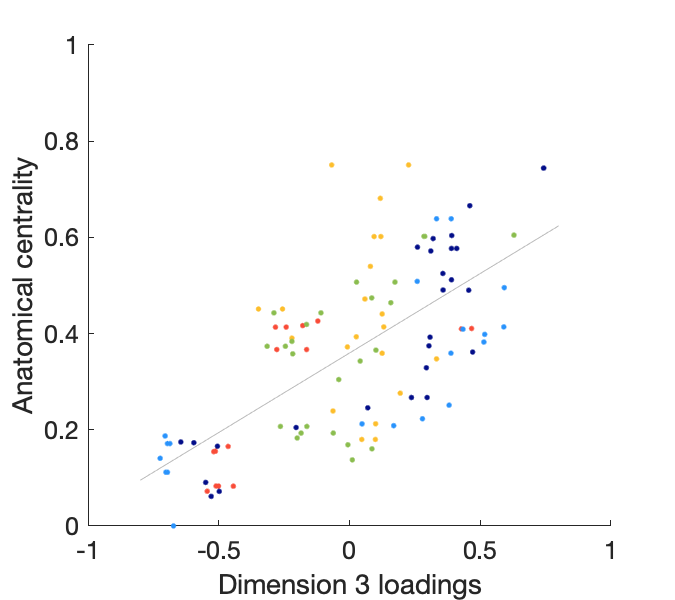


Figure S6. Gradient 3 loadings of the *replication sample* also correlated positively with anatomical centrality (*r* = 0.681, *p* < 0.001).

Figure S7. We tested robustness of the three-dimensional solution of MDS. The original analysis was based on 109 seeds and retained global signal regression (GSR+) (boxed, middle row). We varied these parameters by removing global signal regression from preprocessing (GSR-; top row) and by sampling 264 seeds across the cortex per^3^ (bottom row). Across all three sets of parameters, a three-dimensional solution consistently led to substantial reduction in stress and gain in explained variance. A) Stress was plotted as a function of the number of estimated dimensions. Lower stress indicates better fit. The scree plot of normalized stress had an “elbow” when dimensionality was at 3, since further addition of dimensions did not substantially reduce normalized stress. Dashed line indicates normalized stress of 0.05. B) DAF was plotted as a function of the number of estimated dimensions. Higher DAF indicates better fit. Dashed line indicates DAF of 0.98.

Figure S8. We tested robustness of the circumplex structure of MDS output. The original analysis was based on 109 seeds and retained global signal regression (GSR+) (boxed, middle row). We varied these parameters by removing global signal regression from preprocessing (GSR-; top row) and by sampling 264 seeds across the cortex per^3^ (bottom row). Across both sets of GSR+ solutions (bottom two rows), scatterplots in coordinate space consistently revealed a circumplex-like shape. The GSR- condition (top row) showed decreased power in detecting circumplexity. Each point in the scatterplot represents a connectivity map. Dimensions 1-3 are plotted pairwise in two-dimensional coordinate space to facilitate interpretation.

Figure S9. The anterior commissure (red dot), with MNI coordinates (0, 0, 0), was used as a proxy for the center point of the brain since it is approximately equidistant from the most distal points of the cerebrum, and approximately occupies the middle point along the anterior-posterior axis of the medial limbic ring consisting of the cingulate cortex and medial orbitofrontal cortex.

**

*Figure S10. When functional connectivity maps were thresholded at z > 0.2, both goodness-of-fit estimates were still highly replicable.*

**

*Figure S11. When functional connectivity maps were thresholded at z > 0.2, the circumplex-like structure was retained*.

*Figure S12. When functional connectivity maps were thresholded at z > 0.2 and z < -0.2, both goodness-of-fit estimates were still highly replicable.*

**

*Figure S13. When functional connectivity maps were thresholded at z > 0.2 and z < -0.2, the circumplex-like structure was retained*.

Table S1. Literature-based seeds.

| **Major Domain** | **Network Name** | **Seed** | | | | | |
| --- | --- | --- | --- | --- | --- | --- | --- |
|  |  | **Hemisphere** | **Region** | **Reference** | **MNI Coordinates** | | |
|  |  |  |  |  | **x** | **y** | **z** |
| **Attention** | **Dorsal Attention** | L | frontal eye field | Fox et al. (2006) | -24 | -21 | 49 |
|  |  | L | frontal eye field | Vincent et al. (2008) | -26 | -8 | 50 |
|  |  | L | frontal eye field | Yeo et al. (2011) | -24 | 2 | 58 |
|  |  | L | intraparietal sulcus | Fox et al. (2006) | -28 | -66 | 56 |
|  |  | L | middle temporal motion area | Vincent et al. (2008) | -46 | -70 | -2 |
|  |  | L | premotor cortex | Yeo et al. (2011) | -50 | 10 | 28 |
|  |  | L | superior parietal lobule | Vincent et al. (2008) | -28 | -52 | 58 |
|  |  | L | superior parietal lobule | Yeo et al. (2011) | -24 | -60 | 64 |
|  |  | R | frontal eye field | Fox et al. (2006) | 25 | -20 | 49 |
|  |  | R | frontal eye field | Vincent et al. (2008) | 28 | -8 | 50 |
|  |  | R | frontal eye field | Yeo et al. (2011) | 24 | 2 | 58 |
|  |  | R | intraparietal sulcus | Fox et al. (2006) | 27 | -67 | 57 |
|  |  | R | middle temporal motion area | Vincent et al. (2008) | 46 | -70 | -4 |
|  |  | R | premotor cortex | Yeo et al. (2011) | 50 | 10 | 28 |
|  |  | R | superior parietal lobule | Vincent et al. (2008) | 24 | -56 | 56 |
|  |  | R | superior parietal lobule | Yeo et al. (2011) | 24 | -60 | 64 |
| **Default** | **Amygdala affiliation** | L | medial amygdala | Bickart et al. (2012) | -14 | -4 | -20 |
|  |  | R | medial amygdala | Bickart et al. (2012) | 14 | -4 | -20 |
|  | **Default** | L | anterior medial prefrontal cortex | Andrews-Hanna et al. (2010) | -6 | 52 | -2 |
|  |  | L | hippocampus | Andrews-Hanna et al. (2010) | -22 | -20 | -26 |
|  |  | L | posterior cingulate cortex | Andrews-Hanna et al. (2010) | -8 | -56 | 26 |
|  |  | L | retrosplenial cortex | Andrews-Hanna et al. (2010) | -14 | -52 | 8 |
|  |  | L | temporo-parietal junction | Andrews-Hanna et al. (2010) | -54 | -54 | 28 |
|  |  | R | hippocampus | Andrews-Hanna et al. (2010) | 22 | -20 | -26 |
|  |  | R | retrosplenial cortex | Andrews-Hanna et al. (2010) | 14 | -52 | 8 |
|  |  | R | temporo-parietal junction | Andrews-Hanna et al. (2010) | 54 | -54 | 28 |
|  |  | M | dorsomedial prefrontal cortex | Andrews-Hanna et al. (2010) | 0 | 52 | 26 |
|  |  | M | dorsomedial prefrontal cortex | Yeo et al. (2011) | 0 | 50 | 24 |
|  |  | M | posterior cingulate cortex | Yeo et al. (2011) | 0 | -64 | 40 |
|  |  | M | ventromedial prefrontal cortex | Andrews-Hanna et al. (2010) | 0 | 26 | 18 |
|  | **Language** | L | middle temporal gyrus | Turken & Dronkers (2011) | -64 | -32 | -6 |
|  |  | L | pars triangularis | Turken & Dronkers (2011) | -55 | 34 | 3 |
|  |  | L | pars triangularis | Tomasi & Volkow (2012) | -51 | 27 | 18 |
|  |  | L | superior temporal gyrus | Tomasi & Volkow (2012) | -51 | -51 | 30 |
|  | **Mentalizing** | L | dorsomedial prefrontal cortex | Baetens et al. (2014) | -10 | 58 | 24 |
|  |  | L | precuneus | Baetens et al. (2014) | -4 | -52 | 30 |
|  |  | L | ventromedial prefrontal cortex | Baetens et al. (2014) | -2 | 44 | -12 |
| **Executive** | **Executive** | L | anterior intraparietal lobule | Vincent et al. (2008) | -52 | -50 | 46 |
|  |  | L | anterior intraparietal lobule | Yeo et al. (2011) | -50 | -40 | 48 |
|  |  | L | anterior prefrontal cortex | Vincent et al. (2008) | -36 | 58 | 10 |
|  |  | L | dorsolateral prefrontal cortex | Seeley et al. (2007) | -42 | 34 | 20 |
|  |  | L | dorsolateral prefrontal cortex | Vincent et al. (2008) | -50 | 20 | 34 |
|  |  | L | dorsolateral prefrontal cortex | Yeo et al. (2011) | -50 | 28 | 24 |
|  |  | L | lateral parietal lobe | Seeley et al. (2007) | -42 | -50 | 48 |
|  |  | R | anterior intraparietal lobule | Vincent et al. (2008) | 52 | -46 | 46 |
|  |  | R | anterior intraparietal lobule | Yeo et al. (2011) | 50 | -40 | 48 |
|  |  | R | anterior prefrontal cortex | Vincent et al. (2008) | 34 | 52 | 10 |
|  |  | R | dorsolateral prefrontal cortex | Seeley et al. (2007) | 46 | 36 | 18 |
|  |  | R | dorsolateral prefrontal cortex | Vincent et al. (2008) | 46 | 14 | 44 |
|  |  | R | dorsolateral prefrontal cortex | Yeo et al. (2011) | 50 | 28 | 24 |
|  |  | R | lateral parietal lobe | Seeley et al. (2007) | 46 | -54 | 42 |
|  |  | M | medial superior frontal gyrus | Yeo et al. (2011) | 0 | 28 | 44 |
|  | **Multiple-demand** | L | anterior insula | Fedorenko et al. (2013) | -35 | 18 | 3 |
|  |  | L | inferior frontal sulcus | Fedorenko et al. (2013) | -41 | 23 | 29 |
|  |  | L | intraparietal sulcus | Fedorenko et al. (2013) | -37 | -56 | 41 |
|  |  | L | rostral prefrontal cortex | Fedorenko et al. (2013) | -21 | 43 | -10 |
|  |  | R | anterior insula | Fedorenko et al. (2013) | 35 | 18 | 3 |
|  |  | R | inferior frontal sulcus | Fedorenko et al. (2013) | 41 | 23 | 29 |
|  |  | R | intraparietal sulcus | Fedorenko et al. (2013) | 37 | -56 | 41 |
|  |  | R | rostral prefrontal cortex | Fedorenko et al. (2013) | 21 | 43 | 10 |
|  |  | M | anterior cingulate cortex | Fedorenko et al. (2013) | 0 | 31 | 24 |
|  |  | M | presupplementary motor area | Fedorenko et al. (2013) | 0 | 18 | 50 |
| **Exteroceptive** | **Amygdala perception** | L | ventrolateral amygdala | Bickart et al. (2012) | -28 | -4 | -22 |
|  |  | R | ventrolateral amygdala | Bickart et al. (2012) | 28 | -4 | -22 |
|  | **Auditory** | L | superior temporal gyrus | De Luca et al. (2006) | -56 | -19 | 8 |
|  |  | R | Heschl's gryus | Krienen & Buckner (2009) | 46 | -18 | 8 |
|  |  | R | primary auditory cortex | Andoh et al. (2015) | 58 | -18 | 8 |
|  |  | R | superior temporal gyrus | Andoh et al. (2015) | 57 | -11 | -3 |
|  | **Sensorimotor** | L | postcentral gyrus | Vahdat et al. (2011) | -36 | -38 | 58 |
|  |  | L | precentral gyrus | Krienen & Buckner (2009) | -42 | -24 | 60 |
|  |  | L | premotor cortex | Vahdat et al. (2011) | -26 | -22 | 66 |
|  |  | R | postcentral gyrus | Andoh et al. (2015) | 49 | -25 | 56 |
|  |  | R | precentral gyrus | Andoh et al. (2015) | 1 | -25 | 53 |
|  |  | R | precentral gyrus | Krienen & Buckner (2009) | 42 | -24 | 6 |
|  |  | M | precentral gyrus | Yeo et al. (2011) | 0 | -26 | 58 |
|  | **Visual** | L | middle occipital gyrus | De Luca et al. (2006) | -30 | -93 | 17 |
|  |  | L | occipital fusiform gyrus | Yeo et al. (2011) | -28 | -76 | -14 |
|  |  | L | primary visual cortex | Krienen & Buckner (2009) | -4 | -88 | 2 |
|  |  | R | lingual gyrus | De Luca et al. (2006) | 6 | -80 | -8 |
|  |  | R | occipital fusiform gyrus | De Luca et al. (2006) | 24 | -80 | -17 |
|  |  | R | occipital fusiform gyrus | Yeo et al. (2011) | 28 | -76 | -14 |
|  |  | R | primary visual cortex | Krienen & Buckner (2009) | 4 | -88 | 2 |
| **Salience** | **Amygdala aversion** | L | dorsal amygdala | Bickart et al. (2012) | -22 | -4 | -12 |
|  |  | R | dorsal amygdala | Bickart et al. (2012) | 22 | -4 | -12 |
|  | **Cingulo-opercular** | L | anterior insula | Dosenbach et al. (2007) | -36 | 17 | 3 |
|  |  | L | anterior prefrontal cortex | Dosenbach et al. (2007) | -28 | 53 | 16 |
|  |  | L | dorsal anterior cingulate cortex | Dosenbach et al. (2007) | -2 | 7 | 50 |
|  |  | R | anterior insula | Dosenbach et al. (2007) | 38 | 19 | 0 |
|  |  | R | anterior prefrontal cortex | Dosenbach et al. (2007) | 28 | 51 | 25 |
|  | **Multimodal** | L | anterior insula | Sepulcre et al. (2012) | -50 | 3 | -7 |
|  |  | L | dorsal anterior cingulate cortex | Sepulcre et al. (2012) | -6 | 2 | 48 |
|  |  | L | dorsolateral prefrontal cortex | Sepulcre et al. (2012) | -34 | 43 | 25 |
|  |  | L | lateral occipitotemporal | Sepulcre et al. (2012) | -50 | -61 | 1 |
|  |  | L | pars operculum | Sepulcre et al. (2012) | -58 | -21 | 25 |
|  |  | L | superior parietal lobule | Sepulcre et al. (2012) | -18 | -45 | 57 |
|  | **Salience** | R | caudal anterior cingulate cortex | Seeley et al. (2007) | 8 | 18 | 34 |
|  |  | R | dorsal anterior insula | Touroutoglou et al. (2012) | 36 | 21 | 1 |
|  |  | R | orbital frontal insula | Seeley et al. (2007) | 38 | 26 | -10 |
|  |  | R | ventral anterior insula | Touroutoglou et al. (2012) | 28 | 17 | -5 |
|  | **Ventral Attention** | L | anterior insula | Yeo et al. (2011) | -40 | 12 | -4 |
|  |  | L | anterior intraparietal sulcus | Corbetta et al. (2000) | -25 | -61 | 50 |
|  |  | L | posterior intraparietal sulcus | Corbetta et al. (2000) | -25 | -71 | 53 |
|  |  | L | supramarginal gyrus | Yeo et al. (2011) | -60 | -30 | 28 |
|  |  | L | ventral intraparietal sulcus | Corbetta et al. (2000) | -23 | -71 | 34 |
|  |  | R | anterior insula | Yeo et al. (2011) | 40 | 12 | -4 |
|  |  | R | anterior intraparietal sulcus | Corbetta et al. (2000) | 26 | -64 | 58 |
|  |  | R | posterior intraparietal sulcus | Corbetta et al. (2000) | 20 | -70 | 58 |
|  |  | R | supramarginal gyrus | Yeo et al. (2011) | 60 | -30 | 28 |
|  |  | R | ventral intraparietal sulcus | Corbetta et al. (2000) | 28 | -74 | 21 |
| Note: Hemisphere can be left (L), right (R) or medial (M). Medial seeds have an x-coordinate of 0. | | | | | | | |

**References**

1 Xia, M., Wang, J. & He, Y. BrainNet Viewer: a network visualization tool for human brain connectomics. *PLoS One* **8**, e68910, doi:10.1371/journal.pone.0068910 (2013).

2 Van Essen, D. C. *et al.* An integrated software suite for surface-based analyses of cerebral cortex. *J Am Med Inform Assoc* **8**, 443-459 (2001).

3 Power, J. D. *et al.* Functional network organization of the human brain. *Neuron* **72**, 665-678, doi:10.1016/j.neuron.2011.09.006 (2011).
